# Pulmonary Function Prediction Method Based on Convolutional Surface Modeling and Computational Fluid Dynamics Simulation

**DOI:** 10.3390/healthcare13172196

**Published:** 2025-09-02

**Authors:** Xianhui Lian, Tianliang Hu, Songhua Ma, Dedong Ma

**Affiliations:** 1 School of Mechanical Engineering, Shandong University, Jinan 250061, China; lianxh2019@mail.sdu.edu.cn (X.L.);; 2 Key Laboratory of High Efficiency and Clean Mechanical Manufacture at Shandong University, Ministry of Education, Jinan 250061, China; 3 National Demonstration Center for Experimental Mechanical Engineering Education, Jinan 250061, China

**Keywords:** pulmonary function prediction, convolutional surface modeling, CFD simulation, active breathing

## Abstract

**Purpose:** The pulmonary function test holds significant clinical value in assessing the severity, prognosis, and treatment efficacy of respiratory diseases. However, the test is limited by patient compliance, thereby limiting its practical application. Moreover, it only reflects the current state of the patient and cannot directly indicate future health trends or prognosis. Computational fluid dynamics (CFD), combined with airway models built from medical image data, can assist in analyzing a patient’s ventilation function, thus addressing the aforementioned issues. However, current airway models have shortcomings in accurately representing the structural features of a patient’s airway. Additionally, these models exhibit geometric defects such as low smoothness, topological errors, and noise, which further reduce their usability. This study generates airway skeletons based on CT data and, in combination with convolutional surface technology, proposes an individualized airway modeling method to solve these deficiencies. This study also provides a method for predicting a patient’s lung function based on the constructed airway model and using CFD simulation technology. This study also explores the application of this method in preoperative prediction of the required extent of airway expansion for patients with large airway stenosis. **Methods:** Based on airway skeleton data extracted from patient CT images, a personalized airway model is constructed using convolutional surface technology. The airway model is simulated according to the patient’s clinical statistical values of pulmonary function to obtain airway simulation data. Finally, a regression equation is constructed between the patient’s measured pulmonary function values and the airway simulation data to predict the patient’s pulmonary function values based on the airway simulation data. **Results:** To preliminarily demonstrate the above method, this study used the prediction of FEV1 in patients with large airway stenosis as an example for a proof-of-concept study. A linear regression model was constructed between the outlet flow rate from the simulation of the stenosed airway and the patient’s measured FEV1 values. The linear regression model achieved a prediction result of RMSE = 0.0246 and R^2^ = 0.9822 for the test set. Additionally, preoperative predictions were made for the degree of airway dilation needed for patients with large airway stenosis. According to the linear regression model, the proportion of airway radius expansion required at the stenotic position to achieve normal FEV1 was calculated as 72.86%. **Conclusions:** This study provides a method for predicting patient pulmonary function based on CFD simulation technology and convolutional surface technology. This approach addresses, to some extent, the limitations in pulmonary function testing and accuracy caused by patient compliance. Meanwhile, this study provides a method for preoperative evaluation of airway dilation therapy.

## 1. Introduction

The respiratory system is the internal organ system of the human body that has the closest contact with the external environment. Through the respiratory system, the human body takes in oxygen from the external air and expels carbon dioxide via the bloodstream, maintaining the body’s basic metabolic balance and playing a crucial role in the normal life activities of the human body [1,2]. However, as of 2019, chronic obstructive pulmonary disease (COPD) and lower respiratory tract infections have become some of the most prevalent diseases globally [3]. Respiratory diseases not only seriously threaten human health but also increase the global socio-economic burden [4,5,6]. Pulmonary function tests, a commonly used method for assessing respiratory diseases, are crucial for determining disease severity, evaluating treatment efficacy, and predicting disease prognosis [7,8,9,10]. However, there are several issues with pulmonary function tests in clinical practice:

(1) Pulmonary function tests require patients’ cooperation. The accuracy of their results is affected by patients’ compliance. For patients with severe respiratory distress, cognitive impairment, or vocal cord edema, pulmonary function tests are not suitable for obtaining their pulmonary function data. Additionally, patients experiencing coughing, shortness of breath, or high anxiety tend to have reduced compliance, which leads to decreased accuracy and consistency of test outcomes, as well as lower work efficiency of clinicians [11,12,13,14].
(2) Pulmonary function tests can only reflect patients’ current lung function status. For patients with airway tumor compression or other airway stenoses, they cannot predict patients’ future lung function based on changes in tumor size or the degree of airway narrowing. Additionally, they cannot provide preoperative predictions of lung function after airway expansion surgery.

Therefore, it is necessary to acquire patients’ lung function characteristics through methods other than pulmonary function tests.

In addition to pulmonary function tests, imaging examinations are another commonly used approach for clinical diagnosis of respiratory diseases, and chest CT scans are among the most common [15,16,17,18]. Pulmonary function tests focus on the ventilatory function of the respiratory system, while CT scans focus on its anatomical structure. In recent years, with the advancement of medical technology and the rapid development of computer technology, computational fluid dynamics (CFD) has been increasingly integrated with respiratory imaging data. This integration aims to build three-dimensional (3D) airway models, which help explore the ventilatory characteristics of the respiratory system [19]. Using a simplified upper airway model, Wen-I Li demonstrated numerical simulation results of the solid aerosol depolymerization phenomenon [20]. K.W. Stapleton demonstrated the impact of different turbulence models on airway simulation results [21]. Zhe Zhang conducted detailed numerical simulations of airflow and particle transport/deposition in the airways of tuberculosis patients using a 16-generation airway model [22]. L.L.X. Augusto used an airway model to compare CFD analyses of particle deposition in human lungs during inhalation, exhalation, and breath-holding. The objective was to validate the medical recommendation of holding breath after inhaling aerosols from nebulizers and inhalers [23]. Fouad Farkhadnia focused on a symmetric trifurcation lung airway model, evaluating and comparing airflow patterns and particle transport/deposition in healthy and chronic bronchitis airways under three breathing modes: rest, light activity, and moderate exercise [24]. Geng Tian and A.F. Tena developed simplified respiratory system models and conducted simulations of inhalable particle deposition [25,26]. Jinxiang Xi developed a CFD-fractal airway model to assess the feasibility of detecting the presence and progression of lung diseases [27]. Wenwen Zhang used a planar symmetric airway model to study particle deposition in asthmatic children, investigating the biological pathogenesis of childhood asthma [28].

These models have contributed significantly to their respective fields but have limitations in representing patients’ airway structural characteristics. Specifically, they insufficiently account for actual airway geometry, including airway asymmetry and individual differences. Airways vary among different individuals, and simulation results should also differ for the same individual at different disease stages. Using idealized and simplified airway models for simulations simplifies the analysis but reduces the accuracy of results.

Using the commercial software Mimics to extract airway models has become one of the common methods for clinicians to obtain airway models [29,30]. This method enables the automatic or semi-automatic 3D reconstruction of airway models based on actual imaging data, and the constructed models are sufficient for visual diagnosis. However, the extracted models often have geometric defects such as protrusions, pits, disconnected holes, and excess debris, leading to low smoothness, topological errors, and noise. These defects further affect the overall quality of the models, reduce their usability, and prevent their direct application in further CFD simulation analysis.

The current challenge is to construct personalized airway models usable for pulmonary ventilation simulations. Bloomenthal and Shoemake proposed a convolution surface technique [31]. As a type of implicit surface, convolution surfaces hold distinct advantages for 3D surface modeling. Implicit surfaces, for one, excel at intersection operations—this ensures continuity between surfaces. For another, convolution surfaces themselves boast unique merits: shape definition via a skeleton, smoothness retention even with an unsmooth skeleton, strong mixing capabilities, and simple, efficient application [32]. They are particularly effective for modeling complex tubular structures. The human airway, similar to a tree crown, is a tubular structure with complex branching features; J. Andreas Bærentzen used the convolution surface technique to construct a 3D model of a tree crown and verified the effectiveness of this technique [33].

Additionally, the convolution surface technique has been applied in the medical field. Steffen Oeltze and Guillaume Pizaine attempted visualization modeling of airways and blood vessels using the convolution surface technique, achieving good results [34,35]. Domagoj Bošnjak constructed vascular models with hybrid convolution surfaces, simulated the blood flow of generalized Newtonian fluids in the human aorta, and demonstrated these models’ applicability in practical scenarios [36]. This study employs the convolution surface technique to address insufficient structural representation and existing geometric defects of airway models. The skeleton of the convolution surfaces is derived from CT data, which have realistic and personalized features to accurately represent the airway structure of a patient. Moreover, such convolution surfaces based on real data enable the construction of airway models for simulating pulmonary ventilation function.

In summary, while pulmonary function tests can be used to assess the ventilatory function of the respiratory system, their accuracy is limited by patient compliance, and they have practical operational limitations. Additionally, pulmonary function tests only reflect a patient’s current state and cannot directly predict or evaluate the patient’s post-treatment prognostic status. CFD technology can assist in analyzing a patient’s respiratory ventilation function using airway models based on CT data. However, the models currently used for simulations are insufficient in accurately representing patients’ airway structural characteristics and have geometric defects. Convolution surface technology, which uses real-data-derived airway skeletons, can address the shortcomings of existing models, enabling the construction of personalized airway models for simulating respiratory ventilation functions. This study employs convolution surface technology to construct patients’ personalized airway models and analyzes the relationship between airway simulation data and actual measured pulmonary function values. It also explores the clinical feasibility of predicting lung function by integrating imaging data with CFD analysis. It also investigates the preoperative prediction of the required degree of airway dilation for patients with large airway stenosis—with the aim of helping clinicians develop more personalized surgical plans, improving patients’ overall treatment outcomes, and exploring the feasibility of pulmonary function prediction across different disease stages.

The subsequent chapters of this study are organized as follows: Section 2 provides a detailed description of the research methodology, including model construction, model simulation settings, and pulmonary function prediction; Section 3 presents the core experimental results, including the development and validation of the FEV1 prediction model, flow field characteristics in typical patients, and preoperative evaluation of airway dilation surgery; Section 4 conducts in-depth analysis from three perspectives—clinical significance, application value, and limitations—clarifies the research positioning, and defines future research directions; finally, Section 5 summarizes the core contributions of this study.

## 2. Methods

### 2.1. Overview

To address the limitations in pulmonary function testing and inaccuracies in its results caused by patient compliance constraints, this study combines CFD simulation technology with CT imaging data to analyze respiratory ventilation function. Its main content is divided into three parts: model construction, model simulation settings, and simulation-based pulmonary function prediction.

**Figure 1:**
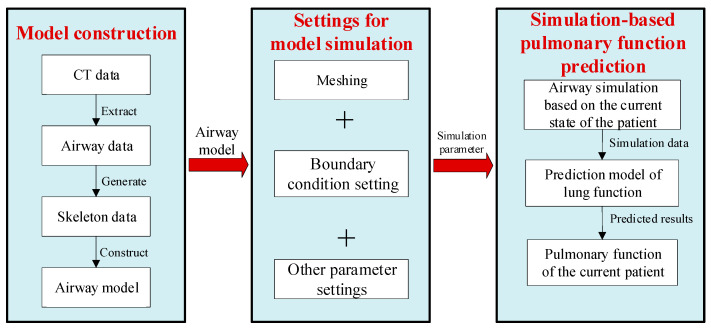
The workflow for the simulation-based pulmonary function prediction method.

As shown in Figure 1, in this study, airway data (the .stl data from Mimics) were first extracted from the patient’s CT scans. Next, skeleton data (include the centerline and radius data) were generated based on the airway centerline within the airway data. Using the skeleton data, the convolution surface technique was employed to construct airway models, which include both the stenotic airway model representing the patient’s current state and the normal airway model representing the expanded state at the stenotic sites. The constructed airway models are of high quality and are designed to strive to reflect the real information of the patient’s airway as accurately as possible. They not only enable more precise representation of the patient’s airway structural characteristics but also facilitate subsequent mesh generation and simulation.

After constructing the airway model, an appropriate mesh, boundary conditions, and fluid dynamics model were selected for CFD calculations.

Based on the simulation results and measured pulmonary function data of different patients, a binary or multivariate mapping relationship was established between simulation data (e.g., cross-sectional volume flow rate, cross-sectional pressure, pressure drop) and measured pulmonary function data (e.g., FEV1, FVC, PEF). This is referred to as the pulmonary function prediction model. As shown in Figure 1, the model takes the simulation results of the patient’s airway model as input and the patient’s pulmonary function as output, addressing the problems caused by patient compliance constraints. Furthermore, the pulmonary function prediction model can also take the simulation results of the patient’s airway model (after changes in airway stenosis severity) as input to predict the pulmonary function of airways with different stenosis severities. This enables preoperative prediction and assessment of the required airway expansion extent for patients with large airway stenosis undergoing airway expansion.

### 2.2. Model Construction

This section provides a detailed introduction to the model establishment method described in Section 2.1, using Patient A from 6 patients with large airway stenosis admitted to the Department of Respiratory Medicine, Qilu Hospital of Shandong University, between 1 January 2023 and 28 February 2024. Based on the established airway model, the pulmonary function prediction method is illustrated by taking FEV1 prediction for patients with large airway stenosis as an example. Additionally, the airway model established herein can also be used to predict the required degree of airway dilation for such patients.

This study was approved by the Research Ethics Committee of Qilu Hospital of Shandong University (Study Title: Pulmonary Function Prediction and Intelligent Ventilator Therapy for Patients with Respiratory Diseases Based on Retrospective Analysis; Approval Number: KYLL-202401-044-1). The CT images of the six included patients all exhibit the geometric features of large airway stenosis, and their medical records, pulmonary function data, and CT data during hospitalization were collected. As this was a retrospective study, informed consent was waived.

#### 2.2.1. Extraction of Airway Data

This study aims to establish personalized models for individual patients. To ensure the constructed airway models accurately reflect the 3D characteristics of the patient’s airway and represent the specific features of their airway structure, this study establishes the patient’s airway models based on the patient’s CT imaging data.

For Patient A, the specific steps are as follows:

Step 1: CT data preprocessing. The patient’s pulmonary CT image data (in DICOM format) were first acquired and imported into Mimics 21.0 software.

Step 2: Airway tree extraction. The airway initiation region (at the main tracheal inlet, with a grayscale value of approximately −500 to −800 HU) was manually selected as the seed point. Using the region-growing algorithm, the airway tree was extracted via Mimics software; erroneously extracted small vascular branches were manually removed, retaining the airway tree structure from the main trachea to the 5th–8th generation bronchi.

Step 3: Airway tree surface optimization. The airway tree surface underwent local smoothing and decimation optimization, and the final airway tree model was exported as an .stl file (referred to as airway data).

**Figure 2:**
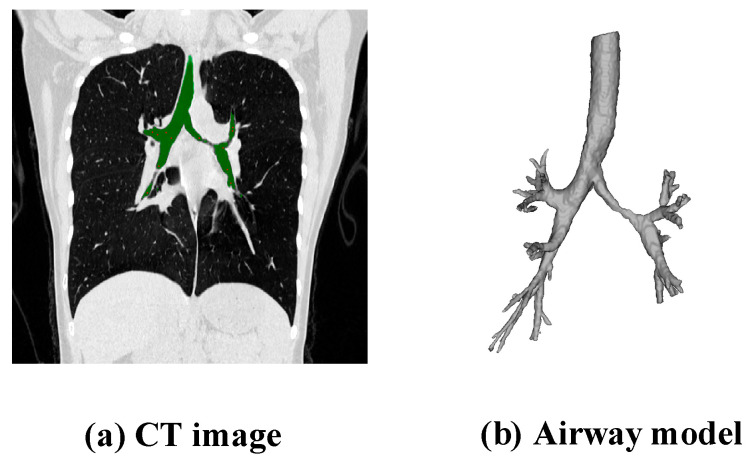
CT image and 3D model of the airway.

As shown in Figure 2, Figure 2a depicts the patient’s CT image, and Figure 2b shows a schematic diagram of the 3D model of the airway data.

#### 2.2.2. Generation of Skeleton Data

**Figure 3:**
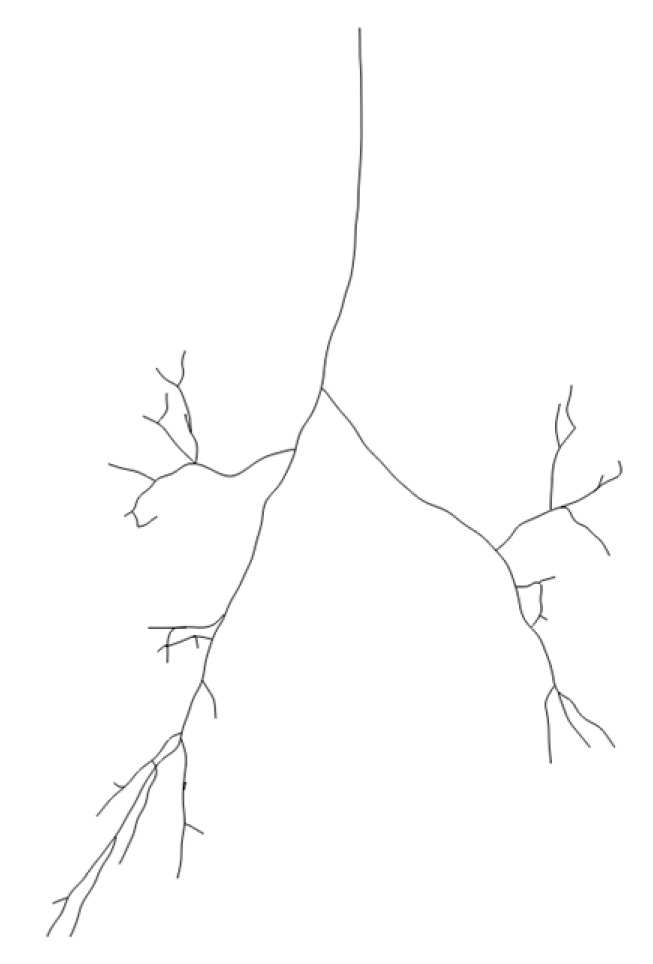
Centerline of airway.

Based on the airway tree extracted in Section 2.2.1, skeleton data were generated through the following steps:

Step 1: Airway centerline extraction. The airway centerline was automatically extracted using the Centerline function in Mimics software and exported as an .igs file. Figure 3 shows a 3D schematic of the airway centerline. The airway tree was graded according to the centerline: the main trachea was defined as Level 0, branches connected to Level 0 as Level 1, branches connected to Level 1 as Level 2, and so on.

Step 2: Equivalent radius calculation. The start and end points of each branch were connected to form its centerline *l_i_*. A perpendicular plane *A_ij_* to the centerline *l_i_* was created through each sampling point *P_ij_*; the area *S_ij_* enclosed by the intersection line of plane *A_ij_* and the airway tree was calculated. The equivalent radius for each sampling point *P_ij_* was determined as *R_ij_ =* $\sqrt{\frac{S_{ij}}{\pi }}$. The radius at branch nodes was optimized via linear transition: if the radius difference between adjacent points at a node exceeded 20%, linear interpolation was used to adjust the transition segment to avoid abrupt radius changes.

Step 3: Skeleton data generation. The nurbsfit function in Matlab R2022b was used for NURBS fitting of the airway centerline. Meanwhile, linear interpolation was performed on the radius of each point to ensure a one-to-one correspondence between the radius data and the centerline sampling points. Finally, the skeleton data were composed of the centerline and radius.

#### 2.2.3. Establishment of the Airway Model

To ensure the generated model can be directly used for simulation, this study employs convolution surface technology for implicit surface modeling of the airway. To accurately characterize the patient’s unique airway structure, the skeleton data (as detailed in Section 2.2.2) is used for convolution surface modeling. The establishment of the airway model primarily consists of two steps: calculation of the skeleton potential function and rendering of the convolution surface from the skeleton.

(1) Calculation of the Skeletal Potential Function

When calculating the skeletal potential function for the convolution surface, it is necessary to accumulate the effect of the skeleton on spatial points through integration. However, traditional kernels have inherent limitations: traditional Gaussian kernels have no definite action boundary (still contributing as *r*→∞), requiring calculation of all skeleton points and leading to redundant computations; traditional linear kernels are simple but their integration results are prone to boundary discretization errors; traditional cubic polynomial kernels have discontinuous first derivatives at *r = R*, easily causing sharp corners at airway bifurcations.

To address these issues, this study selects a truncated quartic polynomial with compact support as the convolution kernel function. This kernel is zero when *r > R*, only considering contributions from the skeleton within the clipping sphere, avoiding invalid computations for skeleton points far from the current tube segment. Meanwhile, as a polynomial, substituting it into the parametric equation of a line segment results in an integrand that remains a polynomial in *t*; analytical integration can thus be used to solve the integral, avoiding volume flow calculation deviations caused by numerical integration. Additionally, the quartic polynomial form of this kernel ensures continuous first derivatives of the potential function at the action boundary (*r = R*), guaranteeing a smooth airway model surface and better matching the transitional morphology of real airway bifurcations.

The form of the truncated quartic polynomial is as follows:

(1) $$f\left(P-Q\right)=\left\{\begin{array}{ll} {\left(1-\frac{r^{2}}{R^{2}}\right)}^{2}, & \;\;r\leq R\\ 0, & \;\;r>R \end{array}\right.$$

where *P* is a point in 3D space, *Q* is an arbitrary point on the linear skeleton, *r = ‖P* − *Q‖* denotes the distance between points *P* and *Q*, and *R* > 0 is the effective radius (only skeleton points within the clipping sphere contribute to *P*).

For a linear segment skeleton, its parametric equation is given by:

(2) $$L\left(t\right)=P_{1}+t\left(P_{2}-P_{1}\right)$$

where *t* ∈ [*t*_1_, *t*_2_] represents the intersection interval of the line segment with the clipping sphere. The potential function of this linear segment skeleton on point *P* is defined as the integral of the kernel function over this interval [37]:

(3) $$F\left(P\right)=l\cdot \int_{t_{1}}^{t_{2}}{\left(1-\frac{{\left\|P-L\left(t\right)\right\|}^{2}}{R^{2}}\right)}^{2}dt$$

where *l = ‖P*_2_ − *P*_1_*‖* is the length of the line segment. To improve computational efficiency during modeling, the integral in Equation (3) is converted into an analytical expansion, with the derivation process as follows:

Define ***d*** *= P*_2_ − *P*_1_ and ***v*** *= P* − *P*_1_. Then:

*P* − *L(t)* = ***v*** − *t*∙***d***

Expand the squared norm *‖P* − *L(t)‖*^2^:

‖*P* − *L(t)*‖^2^ = ‖***v***‖^2^ − 2*t*⋅(***d****⋅**v***) + *t*^2^⋅‖***d***‖^2^

Introduce two auxiliary variables: *a = **d**⋅**v*** and *h = R*^2^ *− ‖**v**‖*^2^. Since *‖**d**‖*^2^ *= l*^2^ and *‖**v**‖*^2^ *= R*^2^ − *h*, substitute these into the expanded norm:

‖*P* − *L(t)*‖^2^ = (*R*^2^ − *h*)-2*at* + *l*^2^*t*^2^

Substitute the above equation into the kernel function in Equation (3) and simplify the integral:

$$\begin{array}{ll} F\left(P\right) & =l\cdot \int_{t_{1}}^{t_{2}}{\left(1-\frac{\left(R^{2}-h\right)-2at+l^{2}t^{2}}{R^{2}}\right)}^{2}dt\\ & =l\cdot \int_{t_{1}}^{t_{2}}{\left(\frac{h+2at-l^{2}t^{2}}{R^{2}}\right)}^{2}dt\\ & =\frac{l}{R^{4}}\int_{t_{1}}^{t_{2}}\left(l^{4}t^{4}-2al^{2}t^{3}+\left(4a^{2}-2hl^{2}\right)t^{2}+4aht+h^{2}\right)dt \end{array}$$

Evaluate the definite integral to obtain the final analytical expansion:

(4) $$\;F\left(P\right)=\frac{l}{R^{4}}\left(\frac{l^{4}}{5}t^{5}-\frac{al^{2}}{2}t^{4}+\frac{\left(4a^{2}-2hl^{2}\right)}{3}t^{3}+2aht^{2}+h^{2}t\right){|}_{t_{1}}^{t_{2}}$$

This final expanded form Equation (4) does not require numerical integration; it can be quickly calculated using only basic arithmetic operations, which effectively improves the efficiency and accuracy of convolution surface construction.

(2) Rendering of the Skeleton Convolution Surface

First, linear transition control was applied to the airway surface radius. Using the skeleton data and kernel function, the skeletal potential function was computed to perform convolution surface modeling. Thus, a stenotic airway model was established, which represents the patient’s current state of large airway stenosis.

Next, the patient’s airway model was compared with airway models of healthy individuals to identify stenotic sites. The radius data at these stenotic sites were expanded to match the patient’s healthy airway level, generating patient-specific normal skeleton data. Based on convolution surface technology, these normal skeleton data were used to construct a normal airway model, representing the patient’s airway in a healthy state.

Additionally, the radius data at the stenotic sites were freely adjusted to values between the patient’s current state and healthy state, thereby establishing a series of transitional airway models.

**Figure 4:**
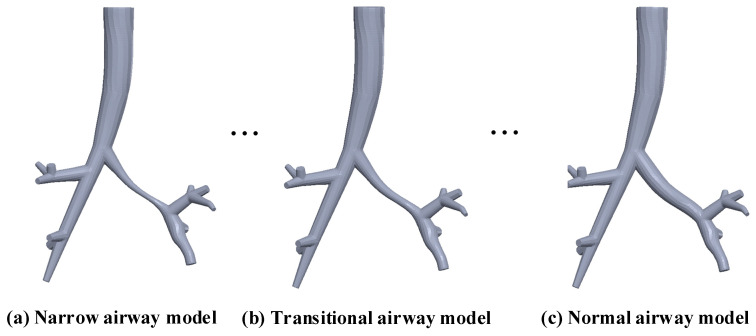
A series of airway models.

Finally, the distal ends of all established airway models were trimmed and optimized to the same length, preparing for subsequent simulations. The final established airway models (stenotic, normal, and transitional) are shown in Figure 4.

### 2.3. Settings for Model Simulation

In model simulation, the setting of appropriate simulation parameters is critical to ensuring the accuracy and validity of results. Proper parameter settings not only effectively reduce the consumption of computational resources but also enhance the reliability of simulation results, laying a solid foundation for subsequent simulations. The simulation process in this study is implemented using the Fluent module in the commercial software Ansys 2022 R2. Based on the airway model established in Section 2.2, this section specifies the settings of simulation parameters, including mesh generation, boundary conditions, material properties, and solver settings.

#### 2.3.1. Mesh Generation

Before the simulation, the entire model was meshed using a tetrahedral meshing method. Given that this study adopted the k-omega turbulence model with enhanced wall treatment (which requires high resolution for the near-wall turbulent boundary layer), an inflation layer was used to achieve refined near-wall meshing. This refinement ensures accurate capture of the velocity gradient, turbulent kinetic energy, and wall shear stress at the airway wall.

**Figure 5:**
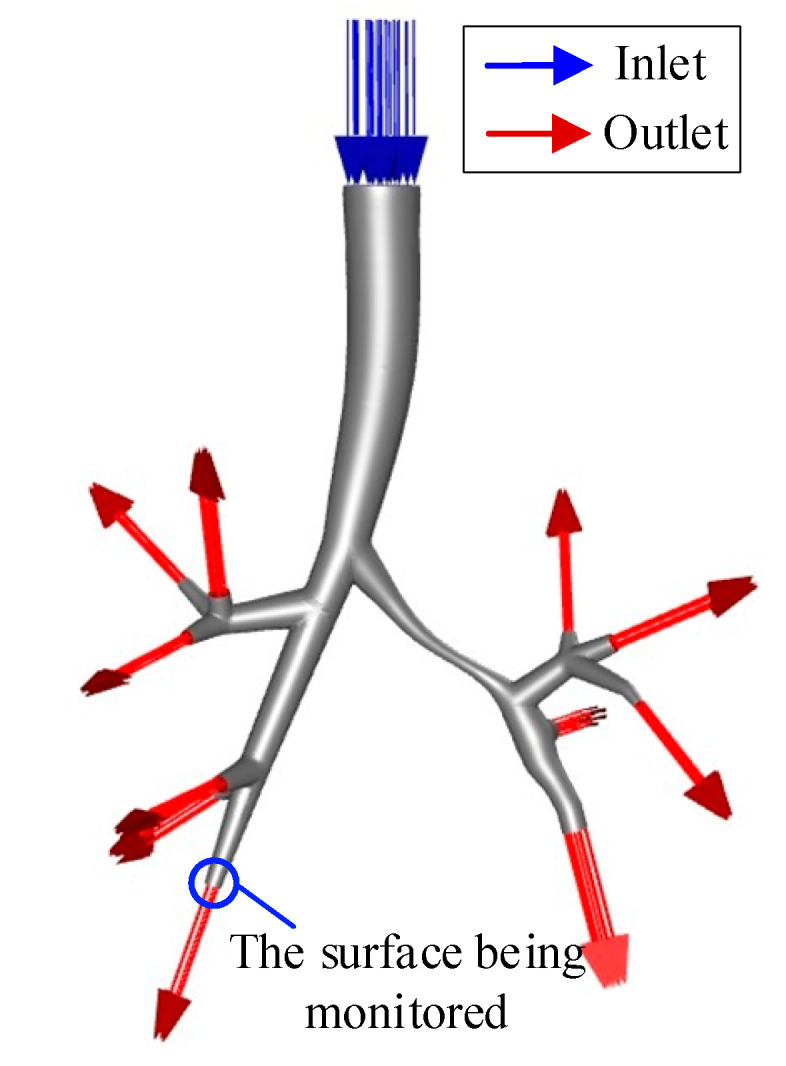
Simulation diagram.

To balance the accuracy of computational results and computational efficiency, it was necessary to determine the optimal mesh count through mesh independence verification. As shown in Figure 5, in the static simulation, the average flow velocity at the large airway outlet was selected as the monitoring index. The inlet velocity corresponded to 7.5 L/min, and the outlet pressure was set to 0 Pa. The impact of different mesh counts on the flow velocity calculation results was tested.

**Figure 6:**
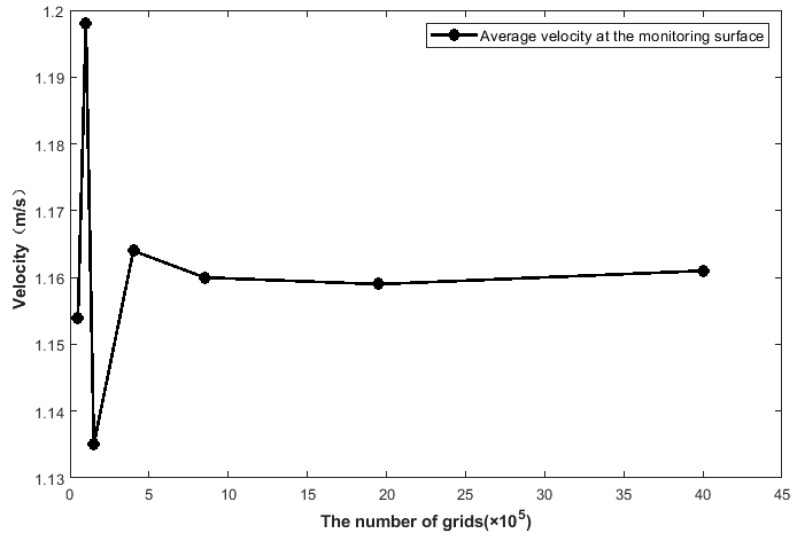
Relationship between the number of grids and the simulation results.

The results are shown in Figure 6: the abscissa represents the number of grids (ranging from 50,000 to 4.2 million), while the ordinate represents the average flow velocity at the monitoring surface, reflecting flow field stability. When the mesh count was small, the flow velocity at the monitoring surface fluctuated significantly between 1.1 and 1.2 m/s. This fluctuation arose due to insufficient mesh quality. Specifically, for complex structures, too low mesh count easily leads to mesh element distortion. This distortion, in turn, led to large deviations in flow velocity calculations and unstable results. When the mesh count increased to 3.2 × 10^5^, the flow velocity stabilized at 1.164 m/s; further increasing the mesh count to 4.0 × 10^6^ resulted in only a 0.3% change in flow velocity, and the computational results no longer varied significantly with increasing mesh count. Considering both simulation accuracy and computational efficiency, 3.2 × 10^5^ was selected as the final mesh count, corresponding to an overall mesh size of 0.75 mm.

In this study, near-wall mesh refinement was achieved using the inflation layer function in Ansys Meshing. The inflation layer was set to 5 layers, the interlayer growth rate was 1.2, and the thickness of the first layer was 0.1 mm. Additionally, to reduce the impact of flow disturbances at inlets and outlets on simulation results, the surface mesh size at all airway inlets and outlets was uniformly set to 0.2 mm, improving the mesh resolution in the boundary regions. The y^+^ distribution along the airway wall was monitored via Fluent’s post-processing: the average y^+^ was 2.3, with a maximum value not exceeding 10 and a minimum of 0.2, which meets the requirements of the k-omega turbulence model for near-wall meshes.

#### 2.3.2. Boundary Condition Setting

Under normal physiological conditions, human breathing is an active process: the contraction and relaxation of the diaphragm and intercostal muscles cause changes in the thoracic cavity, creating a pressure difference between the intrathoracic pressure and the external environment, thereby enabling respiratory ventilation. However, most current simulations have set a velocity inlet or pressure inlet near the oral cavity, which essentially simulates passive ventilation and is inconsistent with the real active breathing process in the human body. The core reason for not adopting active breathing simulations lies in the difficulty of obtaining airflow-related data at the small airway orifices; additionally, conducting invasive experiments on humans is unethical. Consequently, most studies rely on passive ventilation simulations.

To achieve pulmonary function prediction under active breathing, it is necessary to set reasonable boundary conditions, and it is first essential to obtain pressure data at the small airway orifices. Therefore, this study proposes the following two core assumptions:

(1) Lung Segment Function Proportion Assumption

Based on the classical theory of “18-segment lung function proportion”, this study assumes that each lung segment has a fixed proportion of lung function, as follows:

Left upper lobe: Apical posterior segment 6.0%, anterior segment 6.0%, superior lingual segment 4.3%, and inferior lingual segment 4.3%.

Left Lower Lobe: Dorsal segment 6.1%, anteromedial basal segment 6.1%, lateral basal segment 6.1%, and posterior basal segment 6.1%.

Right Upper Lobe: Apical segment 6.9%, posterior segment 6.9%, and anterior segment 6.9%.

Right Middle Lobe: Lateral segment 4.5% and medial segment 4.5%.

Right Lower Lobe: Dorsal segment 4.9%, anterior basal segment 4.9%, medial basal segment 4.9%, lateral basal segment 4.9%, and posterior basal segment 4.9%.

Building on this theory, this study further assumes that the lung function proportion of each segment equals its gas flow proportion. In other words, based on the total flow rate at the large airway outlet, this study allocates the corresponding flow rates to the small airway orifices of different lung segments according to the above proportions. The small airway orifices are set as velocity inlets with proportional flow allocation, while the large airway outlet is set as a zero-pressure outlet. The pressure at the small airway orifices is obtained via simulation.

(2) Assumption of Equal Pressure

For patients with normal small airway function but large airway stenosis, this study hypothesizes that the pressure within the small airways remains unchanged between stenotic and normal large airway conditions. It is critical to clarify that this does not imply uniform pressure distribution across small airways in different lung segments. Instead, it refers to the absence of significant variation in pressure at the same anatomical location of small airways within the same patient, regardless of large airway stenosis. This assumption represents a reasonable simplification proposed to address the lack of direct small airway pressure data, rather than fully replicating physiological reality. The reasonableness of this assumption will be further elaborated by integrating physiological mechanisms, technical limitations, and applicability to the research context.

First, from the perspectives of physiological structure and airflow dynamics, small airways, acting as peripheral pulmonary airways, exhibit relatively stable pressure. Due to their large total cross-sectional area, small airways have low airflow resistance and gentle pressure decay. Under normal physiological conditions, the pressure of small airways is mainly regulated by the elastic recoil force of lung tissue. The core impact of large airway stenosis is confined within the large airways (e.g., increased flow velocity in the stenotic segment and local pressure drop). When pressure fluctuations are transmitted to the small airways, they are attenuated by the buffering effect of small airway branches. Owing to the numerous branches of small airways and a sudden increase in their total cross-sectional area, airflow velocity drops sharply, ultimately causing minimal interference with small airway pressure.

Second, precise measurement of small airway pressure remains technically unachievable with current approaches. Invasive methods (e.g., pressure sensor implantation via bronchoscopy) carry a high risk of patient trauma and face challenges in obtaining ethical approval, whereas non-invasive approaches (e.g., indirect estimation via imaging techniques) lack sufficient predictive precision. Thus, the “equal pressure assumption” serves as a reasonable simplification to address the missing pressure data in small airways, rather than perfectly replicating physiological reality.

Finally, this assumption is strictly limited to patients with normal small airway function. If a patient concurrently has small airway lesions, the pressure stability of small airways is compromised, and thus this assumption no longer applies. For instance, in cases such as congenital large airway stenosis and unilateral large airway tumor compression, patients typically present with large airway abnormalities while maintaining normal small airway function. These are the common clinical scenarios specifically targeted by this study.

Based on the above assumptions, this study obtained the pressure at the small airway orifices through simulation of the patient’s normal airway model. This pressure is then used as the inlet boundary condition for the small airways in the patient’s large airway stenosis model, thereby completing the active breathing simulation.

**Figure 7:**
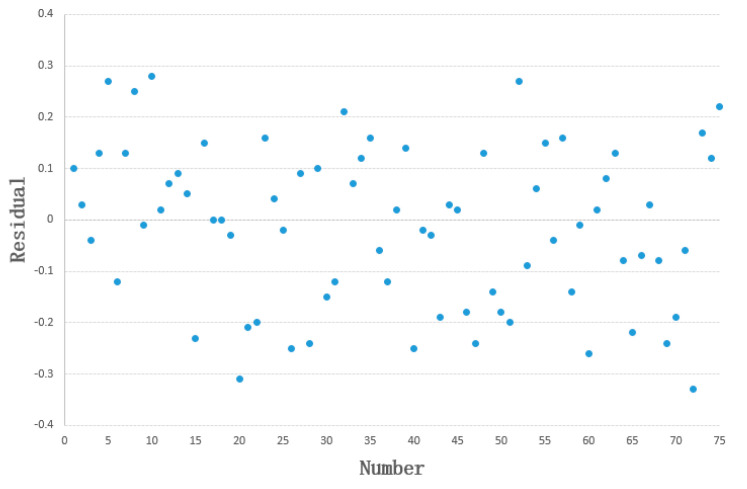
Residual plot of FEV1.

In this study, the outlet flow rate under the patient’s normal state was obtained from the predicted FEV1 values in the built-in reference equation of the Jaeger pulmonary function tester. To ensure the model’s reliability, this reference equation was validated: lung function data from 75 randomly selected healthy individuals were acquired, and a statistical analysis was performed on the measured FEV1 values versus the predicted FEV1 values from the Jaeger pulmonary function tester. The correlation between the two datasets was r = 0.97 (*p* < 0.05), indicating a strong correlation. The residual plot is shown in Figure 7, with a mean residual of 0.0144, a standard deviation of 0.15472, and a mean standard error of 0.01787 (*p* > 0.05). This result demonstrates that there is no significant difference between the predicted and measured values.

Therefore, this study used the FEV1 predicted values from the reference equation of the Jaeger pulmonary function tester as the original data. In accordance with the lung segment function proportion theory, the predicted FEV1 values were proportionally allocated as the velocity inlet boundary conditions for the small airways of the normal model, while the large airway was set as a zero-pressure outlet boundary condition. Through simulation, the inlet pressure at the small airways of the normal model was obtained. Subsequently, this inlet pressure (from the normal model) was applied to the small airways of the stenotic model as the inlet pressure boundary condition; the large airway of the stenotic model was also set as a zero-pressure outlet boundary condition. Finally, the volume flow rate at the large airway outlet was monitored.

#### 2.3.3. Other Parameter Settings

To align with the airflow characteristics of real human breathing, this study selected air at standard atmospheric pressure (101,325 Pa) as the fluid material. Given that all patients in this study breathed normal air without inhaling special gases, the standard air density of 1.204 kg/m^3^ was adopted. The dynamic viscosity was set to 1.81 × 10^−5^ Pa·s—an inherent property of the fluid that is not affected by individual patient parameters. Additionally, the human breathing rate is less than 83.3 m/s (Mach number below 0.3); thus, the airflow during breathing can be regarded as incompressible flow. Due to the complex structure of the airway, collisions between airflow and airway walls during human breathing inevitably generate vortices, which, in turn, cause flow turbulence. Therefore, the fluid flow during breathing was treated as unsteady turbulent flow in this study.

In the simulation, the airway wall was assumed to be rigid with no deformation occurring during breathing. The k-omega turbulence model was adopted, and the SIMPLEC algorithm was employed for pressure-velocity coupling. The gradient format was Least Squares Cell-Based, with pressure interpolation performed using the Second Order scheme. The momentum equation, turbulent kinetic energy, turbulent specific dissipation rate, and energy terms are all discretized using the second-order upwind scheme. To simulate the active exhalation process, the small airway orifices were set as inlets and the large airway orifice was set as an outlet. The convergence criterion was a residual value less than 10^−3^.

### 2.4. Prediction of FEV1 Based on Large Airway Flow Simulation

To predict FEV1 in patients with large airway stenosis using CFD simulation technology, this section defines the input parameters and output indicators of the lung function prediction model and establishes the mapping relationship between them.

The basic information of the patients used in this study is shown in Table 1. All patients exhibited varying degrees of FEV1 reduction, while changes in FVC were relatively minor. This is consistent with the structural characteristics of patients with large airway stenosis. The alveolar function of patients with large airway stenosis is generally intact, which has a minimal effect on vital capacity. However, the stenotic large airway elevates expiratory resistance, leading to a decrease in FEV1.

Based on the model establishment method in Section 2.2 and simulation method in Section 2.3, the two methods were separately applied to the data of the six patients, ultimately obtaining the outlet volume flow rate of each patient’s stenotic airway model. This study conducted statistical analysis on the outlet volume flow rate of the patients’ stenotic models and their measured FEV1 values and established a linear regression model between the two. The linear regression analysis was implemented using SPSS 20, based on the least squares principle. The outlet volume flow rate of the patient’s stenotic model was used as the input of the lung function prediction model, and the patient’s measured FEV1 value was used as the output. By inputting the outlet volume flow rate of the patient’s stenotic airway model, this regression model can predict the patient’s measured FEV1 value. Detailed results are presented in Section 3.

## 3. Results

Based on the FEV1 prediction method proposed in Section 2, this chapter presents the experimental results and core findings, focusing on three parts: the preliminary verification results of the model, flow field visualization of a typical patient, and preoperative evaluation data of airway dilation.

### 3.1. Development and Validation Results of the FEV1 Prediction Model

This study is a proof-of-concept (PoC) research, with its core objective to verify the feasibility of the technical approach: predicting FEV1 via large airway flow field simulation. After rigorous screening, only six eligible patients were included, all of whom had large airway stenosis with normal small airway function. Among them, four patients (coded as A, B, C, D) were used as the training set for preliminary model development, and two patients (coded as E, F) were used as the test set to explore technical feasibility. The specific workflow and results are as follows:

#### 3.1.1. Build a Model Using the Training Set

CFD simulations were performed on the stenotic large airway models of the four patients in the training set to obtain the outlet velocity of the large airways. Based on the cross-sectional area of the large airway outlet, this velocity was converted to a volume flow rate (denoted as a) with a unit consistent with that of FEV1. Meanwhile, the patients’ measured FEV1 values (denoted as b) and the normal predicted FEV1 values (denoted as c) from the built-in reference equation of the Jaeger pulmonary function tester were collected. The summarized data are presented in Table 2.

To eliminate the impact of individual physiological differences (e.g., height, weight) on the absolute value of FEV1, this study selected normalized ratios as the input and output variables of the model. Input variable *x*: a/c (ratio of the simulated outlet volume flow rate to the normal predicted FEV1 value), which reflects the degree of airflow limitation caused by large airway stenosis; output variable *y*: b/c (ratio of the measured FEV1 value to the normal predicted FEV1 value), which reflects the relative decline ratio of lung function.

The least squares method was employed to develop the linear regression model, with the expression:

(5) y=0.8199x−0.089

Tests of model coefficients revealed that both the slope (0.8199, 95% CI: [0.732, 0.908], *p* < 0.05) and the intercept (−0.089, 95% CI: [−0.156, −0.022], *p* < 0.05) were statistically significant. The overall fitting metrics of the training set were RMSE = 0.0246 and R^2^ = 0.9822. The mean residual was 0.003, the residual standard deviation was 0.018, and the residual range was [−0.021, 0.025].

Given the small sample size of the training set, leave-one-out cross-validation (LOOCV) was conducted to preliminarily assess whether the model suffered from severe overfitting. For each iteration, one patient from the training set was excluded as the validation sample, and the remaining three patients were used as a new training set to redevelop the model. This process was repeated four times, after which the average performance metrics were calculated. Results showed that the RMSE range of cross-validation was [0.01, 0.05], and the R^2^ range was [0.98, 0.99]. These values exhibited minimal differences from the metrics of the original training set (RMSE = 0.0246, R^2^ = 0.9822), indicating no severe overfitting of the model within the current small sample size. However, it should be noted that the possibility of “inflated metrics due to accidental sample distribution” cannot be completely ruled out.

**Figure 8:**
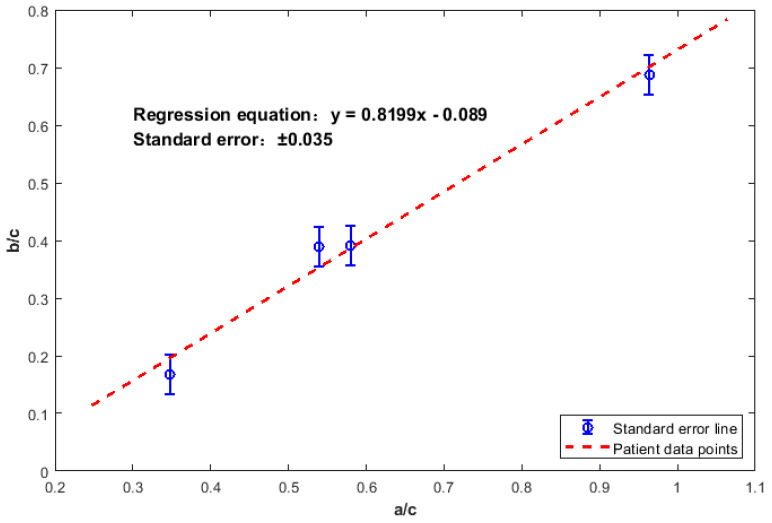
Linear regression equation (with error bars of standard error; error bars represent ±residual standard error = 0.035).

Additionally, as shown in Figure 8, the patient data were randomly distributed on both sides of the regression line. This confirms the rationality of the linear assumption within the current sample size.

#### 3.1.2. Exploring Feasibility Using the Test Set

Next, a preliminary exploration of the model’s predictive performance was conducted using two additional patients, E and F, as the test set. The basic information for these two patients is shown in Table 3. The final results obtained from Equation (5) are shown in Table 4.

The results from the linear regression equation are as follows:

Root Mean Square Error: 0.0424.

Coefficient of Determination: 0.9424.

For the test set patients:

Patient E: FEV1 prediction error is 4.3%.

Patient F: FEV1 prediction error is 8.8%.

This result preliminarily suggests that the technical approach of establishing the correlation between flow field and pulmonary function is feasible and demonstrates that the method has no obvious logical flaws under the current small sample size. However, it is important to emphasize that due to the test set only including two samples, the aforementioned metrics lack statistical representativeness. They can neither confirm the reliability of prediction nor support the model’s clinical generalization ability. This result only provides a technical direction reference for subsequent studies with expanded sample sizes.

### 3.2. Visualization of Flow Field Characteristics

**Figure 9:**
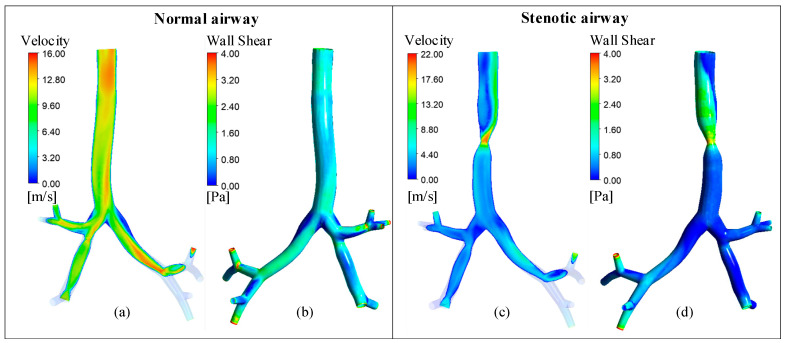
Flow field distribution of a normal airway and a stenotic airway. (**a**) The flow rate of the normal airway; (**b**) The wall shear stress of the normal airway; (**c**) The flow rate of the stenotic airway; (**d**) The wall shear stress of the stenotic airway.

To further verify the rationality of the prediction model, this study selected Patient D (with the greatest deviation of FEV1 from the normal) as the research object and compared the velocity distribution and wall shear stress (WSS) distribution between normal airway model and stenotic airway model. As shown in Figure 9, Figure 9a,b represent the flow field of the normal airway model, while Figure 9c,d represent that of the stenotic airway model.

From the perspective of the overall flow field, the stenotic airway model exhibits distinct characteristics of “abrupt flow velocity change and increased WSS” at the stenotic segment. This shows a sharp contrast to the uniform flow field of the normal airway model. Specifically:

In terms of velocity distribution, as shown in Figure 9a,c, the flow velocity within the large airway of the normal model is uniform, whereas the stenotic model shows a sudden increase in flow velocity at the stenotic segment.

In terms of WSS distribution, as shown in Figure 9b,d, the WSS of the normal model is uniformly distributed along the airway wall, with no localized concentration areas. In contrast, the stenotic model exhibits a unilateral concentrated distribution of WSS.

**Figure 10:**
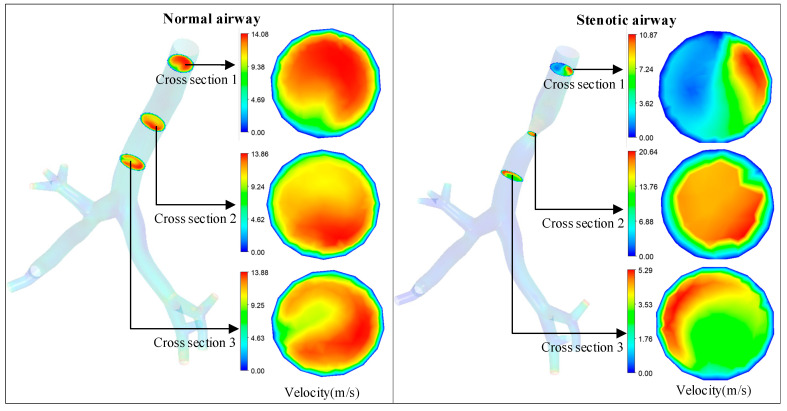
Cross-sectional velocity distribution of a normal airway and a stenotic airway.

Further observation of the flow velocity distribution across airway cross-sections was conducted. As shown in Figure 10, the flow velocity across the normal airway cross-section exhibits a concentric circular pattern, decreasing uniformly from the center to the airway wall. In contrast, the stenotic airway shows an asymmetric velocity distribution across its cross-section, with the high-velocity region concentrated on one side. In particular, for Cross-section 1 of the stenotic airway, the high-velocity region is focused on the stenotic side, and this region highly overlaps with the high-value region of WSS in Figure 9d. This confirms the correlation between velocity concentration and WSS concentration and indirectly verifies that the model’s depiction of airway flow field details aligns with actual physical laws, providing visual support for the rationality of the FEV1 prediction model. The flow velocity at the airway wall in the figures is zero, which is consistent with the “no-slip condition” in fluid mechanics: this condition states that when a viscous fluid flows over a solid wall, no relative sliding occurs between the fluid and the wall; consequently, the flow velocity at the wall is strictly zero, and the velocity in the near-wall region exhibits a gradient distribution along the normal direction.

### 3.3. Preoperative Evaluation Method for Airway Dilation Surgery

Based on the aforementioned preliminarily validated FEV1 prediction model, this study takes Patient E from the test set as an example to explore its application in the preoperative evaluation of airway dilation surgery. Clinical engineers can simulate the stenosis-to-normal transition model and, in conjunction with the guidance of clinicians, predict the required dilation ratio to restore the patient’s FEV1/FVC to the normal range (FEV1/FVC ≥ 70%, correspondingly, FEV1 ≥ 1.869 L/s). The workflow and results are as follows:

First, using the convolution surface modeling method in Section 2.2, a series of transition models for Patient E—from the current large airway stenosis state to the normal state—were constructed. The airway radius at the stenotic site was gradually increased to the normal level, resulting in six airway models with dilation radius ratios (α) of 0%, 20%, 40%, 60%, 80%, and 100%, where $\alpha =\frac{R-R_{2}}{R_{1}-R_{2}}$, *R* is the airway radius after dilation, *R*_2_ is the airway radius at the stenotic site before dilation, and *R*_1_ is the normal airway radius.

Subsequently, in accordance with the simulation methods outlined in Section 2.3 and Section 2.4, the outlet volume flow rate of the large airway was calculated for each transition model. Using the linear regression prediction model, the FEV1 values for each airway model were predicted and fitted. It was found that a dilation ratio α of 72.86% corresponds to an FEV1 of 1.869 L/s.

In summary, under the active exhalation situation, to restore the FEV1/FVC of patient E to above 70%, a dilation ratio α of 72.86% is required. This provides a specific quantitative target for airway dilation surgery.

## 4. Discussion

This chapter centers on the objective data presented in Section 3, focusing on the clinical significance of the results, methodological value, and limitations.

### 4.1. Clinical Significance of the Pulmonary Function Prediction Model

PFT is the core method for diagnosing respiratory diseases, but it is constrained by two key factors: poor patient compliance and patients with contraindications [38,39,40]. For most patients, poor cooperation compromises the accuracy of PFT results; moreover, patients with conditions such as pneumothorax, myocardial infarction, or aortic aneurysm cannot tolerate PFT. These issues result in gaps in pulmonary function assessment for some patients with large airway stenosis. To address this clinical challenge, this study proposes an approach to obtain pulmonary ventilation function characteristics based on CFD simulation technology. This provides a non-invasive, safe assessment pathway for FEV1 in the aforementioned special populations.

From the perspective of technical validation results, this method provides preliminary support for its reliability. The residuals of the training set are randomly distributed without systematic bias, confirming the rationality of the linear assumption. All measured values of the test set fall within the 95% CI, with the FEV1 prediction error lower than 0.15 L/s. Slope and intercept both have statistical significance, providing a basis for the statistical rationality of the method. Compared with traditional pulmonary function prediction methods based on quantitative CT analysis (which have a prediction error range of up to 20% [41,42]), this study has achieved a preliminarily validated prediction error of less than 10%. The narrower error range demonstrates the technical advantages of the proposed method.

Notably, this study is essentially a proof-of-concept research—its core objective is to verify the feasibility of the technical pathway “predicting FEV1 via airway fluid dynamics simulation”, rather than directly providing a mature clinical tool. Although the current results demonstrate exploratory value in two aspects (i.e., offering a non-invasive assessment approach for patients with contraindications and providing a preliminary quantitative reference for airway dilation surgery), all conclusions are based on a small sample size. Thus, the clinical validity of the method still requires further verification through large-sample studies in the future.

Despite the preliminary validation of the technical rationality of this method, there are three potential sources of error:

(1) Sample size limitation: Only six samples were included, which did not cover all subtypes of large airway stenosis. This may lead to prediction bias for special cases (e.g., the relative error of 8.8% for Patient F was slightly higher, possibly due to their stenotic location near the right main bronchus).
(2) CFD simulation error: Assumptions such as lung segmental function proportion and the equal pressure assumption for small airways may introduce calculation bias in volume flow rate, indirectly affecting the accuracy of the input variable *x*.
(3) Limitation in variable selection: The model only incorporates volume flow rate and does not account for clinical factors such as patient age and disease duration, which may result in unexplained variation in residuals.

Furthermore, the convolution surface modeling technique used in this study exhibits significant methodological advantages. Compared with traditional Mimics modeling, the airway models constructed in this study have a smooth surface, free from geometric defects (e.g., low smoothness and topological errors) caused by protrusions or disconnected holes, effectively improving the usability of the models for CFD simulation. Meanwhile, the flow field comparative analysis in Figure 9. shows that the stenotic airway exhibits characteristics of increased flow velocity and concentrated WSS, which is fully consistent with the fluid dynamics theory of large airway stenosis. This further supports the physical rationality of the model and provides a reliable modeling method for subsequent similar studies.

### 4.2. Application Value of Preoperative Evaluation in Airway Dilation Therapy

PFT can only assess a patient’s current pulmonary function status; it cannot predict future status or quantify surgical goals for airway dilation. The FEV1 prediction model established in this study precisely fills this gap. It can be directly integrated into preoperative evaluation for airway dilation therapy, improving patient outcomes.

After obtaining a patient’s CT data preoperatively, clinicians can complete airway extraction, convolution surface modeling, and CFD simulation using this method, and then output a correlation curve between “dilation ratio of the stenotic segment and FEV1 improvement”. The entire workflow takes approximately 3–6 h, providing sufficient theoretical basis and clear surgical goals for the planned surgery. For Patient E, the model explicitly calculates that a dilation radius ratio of 72.86% is required to restore FEV1/FVC to the normal range. This provides a precise quantitative target for surgery, avoiding the risks of airway injury due to excessive dilation or insufficient dilation associated with traditional empirical dilation. For complex cases such as multi-segmental stenosis, the model can also generate simulation results for different dilation combination schemes. By comparing the magnitude of FEV1 improvement and analyzing fluid dynamics data (e.g., WSS, flow velocity, and pressure), this model can assist clinicians in selecting the optimal treatment scheme.

The model also plays an important role in preoperative doctor–patient communication and postoperative management. Simulation results can intuitively demonstrate the expected postoperative FEV1 improvement to patients, thereby enhancing their compliance with surgery. During postoperative review, the extent to which the surgery meets the target can be quickly assessed by comparing the postoperative airway model with the preoperative target dilation model. If FEV1 fails to meet expectations, the causes can be traced using simulation results (e.g., whether there is an unforeseen small airway obstruction), facilitating the optimization of rehabilitation plans.

### 4.3. Limitations and Future Works

Although this study demonstrates certain clinical value, it still has three core limitations that need to be viewed objectively and addressed through targeted optimization.

(1) Limitations

- Sample size limitation

Only six patients were included in this study, which gave rise to two key issues. On one hand, high *R*^2^ values carry the risk of numerical inflation. Leave-one-out cross-validation ruled out serious overfitting in the current small sample size, but the possibility that the high indices result from random sample distribution cannot be completely excluded. On the other hand, the generalization ability of the model was limited. The current samples only cover tracheal and main bronchial stenosis, while the prediction performance of the model for special subtypes (e.g., lobar bronchial stenosis, multi-segmental stenosis) remains unknown.

- Operational limitation

The entire workflow takes 3–6 h and relies on high-performance computing equipment, making it unable to meet emergency scenarios like acute airway obstruction. The operation requires clinical engineers to undergo 1–2 days of training to master the workflow, and clinicians cannot perform it independently. This increases the difficulty of popularizing the technology. Due to the limitation of CT data resolution, only approximately the 8th-generation airways can be extracted; it is impossible to construct a complete 23-generation airway model based on actual anatomical data; this structural simplification may compromise simulation accuracy due to simplified airway structures.

- Applicability limitation

It is only applicable to patients with large airway stenosis and normal small airway function and is not suitable for cases complicated by small airway obstruction. The model only incorporates volume flow rate as a single input variable, without considering clinical factors such as patient age and lung compliance. This may lead to unexplained residual variation in predictions.

(2) Future Works

To address the aforementioned limitations, future studies will focus on advancing the following three aspects of optimization:

- Expanding sample size

Collect over 100 eligible patients, covering different ages, genders, stenosis locations, and severities. This will fundamentally resolve the generalization issue caused by small sample sizes.

- Model optimization

Incorporate clinical parameters (e.g., patient age, lung compliance) to establish a multivariable nonlinear regression model, reducing the limitations of the current single-variable model. Conduct parameter sensitivity analysis. Quantify the error transfer coefficients of key parameters (e.g., airway radius, boundary conditions) and identify the parameters that have the greatest impact on prediction results. This will provide a basis for subsequent optimization of model robustness.

- Technical optimization

Develop a one-click clinical toolkit to automate CT preprocessing, airway modeling, and CFD simulation, streamlining the entire process. Deploy cloud-based computing power to eliminate the need for hospitals to purchase dedicated servers, facilitating the implementation of this method in primary hospitals. Utilize higher-resolution imaging data to optimize airway extraction algorithms, gradually enabling the construction of a complete 23-generation airway model and reducing simulation errors caused by structural simplification.

## 5. Conclusions

To address the limitations and accuracy issues due to the constraints of patient compliance during clinical pulmonary function testing, this study proposes a predictive method for assessing lung function based on convolution surface technology and CFD simulation. The main contributions are as follows:

(1) Development of an airway modeling method using convolution surface technology and establishment of an individualized airway model: A novel approach was proposed for airway modeling based on convolution surface technology, using airway skeleton data extracted from patient CT images. The developed personalized airway models retained the structural features of the patient’s airways and addressed the deficiencies and geometric inaccuracies of existing simulation models in representing airway structures.

(2) Proposal of a simulation method for active breathing and provision of reasonable boundary conditions for active breathing simulations: By first establishing a healthy model of the patient and using statistical reference values of lung function to simulate the fluid dynamics data of the healthy model, this study then transferred this simulation data to the patient’s disease model to perform active breathing simulations. This approach solves the problem of inadequate input data for active breathing simulations due to the difficulty in measuring internal fluid dynamics parameters of the human airway.

(3) Proposal of a predictive method for lung function: This study introduced a method to predict patient lung function using convolution surface technology creating personalized airway models. By simulating these models based on clinical statistical values of lung function, this study obtained airway simulation data and established a linear regression equation relating patient lung function values to the simulation data. This study provided a detailed demonstration of the pulmonary function prediction method using the example of predicting FEV1 in patients with large airway stenosis. This method helps to address the limitations and accuracy issues in pulmonary function testing due to patient compliance constraints.

(4) Proposal of a preoperative prediction method for the degree of dilation required in the treatment of large airway stenosis: This study created a series of transitional models from stenotic to normal airway states to explore the degree of dilation needed to achieve a normal FEV1/FVC ratio. This has significant implications for predicting the disease prognosis and preoperative evaluation of airway dilation procedures for patients with large airway stenosis.

## Figures and Tables

**Table 1:** Patient information.

Number	Gender	Age	Height	Weight	FVC		FEV1	
					Measured	Predicted	Measured	Predicted
A	Female	23	166	56	3.74	3.81	2.29	3.33
B	Female	37	147.5	56	2.84	2.66	0.89	2.28
C	Female	68	156.5	47	2.79	2.25	0.73	1.86
D	Female	67	145.3	40	2.29	1.78	0.24	1.44
E	Female	62	162	61.8	2.26	2.67	0.7	2.25
F	Female	70	159	68	1.92	2.31	1.27	1.91

**Table 2:** Patient data of the training set.

Patient ID	a (L/s)	b (L/s)	c (L/s)	a/c	b/c
A	3.210	2.29	3.33	0.964	0.688
B	1.230	0.89	2.28	0.539	0.390
C	1.079	0.73	1.86	0.580	0.392
D	0.500	0.24	1.44	0.347	0.167

Note: a represents the outlet volume flow rate obtained from the simulation. b represents the measured FEV1 value of the patient. c represents the predicted FEV1 value under normal conditions based on the reference equation from the Jaeger pulmonary function tester.

**Table 3:** Patient data of the test set.

Patient ID	a (L/s)	b (L/s)	c (L/s)	a/c	b/c
E	1.135	0.700	2.250	0.504	0.311
F	1.620	1.270	1.910	0.848	0.665

**Table 4:** The predicted and measured results of the test set.

Patient ID	The Predicted Value of b/c		The Measured Value of b/c	The Predicted Value of b	The Measured Value of b
	Value	95% CI			
E	0.325	[0.298, 0.352]	0.311	0.730	0.700
F	0.606	[0.572, 0.640]	0.665	1.158	1.270

## Data Availability

The datasets presented in this article are not readily available because of ethics issues. Requests to access the datasets should be directed to Xianhui Lian.
